# Advances in hematopoietic stem cell transplantation in the Asia-Pacific region: the second report from APBMT 2005–2015

**DOI:** 10.1038/s41409-019-0554-9

**Published:** 2019-05-14

**Authors:** Minako Iida, Yoshihisa Kodera, Anthony Dodds, Aloysius Yew Leng Ho, Ian Nivison-Smith, Mafruha Rumana Akter, Tong Wu, Albert Kwok Wai Lie, Ardeshir Ghavamzadeh, Hyoung Jin Kang, Tee Chuan Ong, Aye Aye Gyi, Tasneem Farzana, Honorata Baylon, Lallindra Gooneratne, Jih-Luh Tang, Udomsak Bunworasate, Van Man Huynh, Alok Srivastava, Shinichiro Okamoto, Yoshiko Atsuta, Registry Committee of the Asia-Pacific Blood and Marrow Transplantation Group (APBMT)

**Affiliations:** 1 Department of Promotion for Blood and Marrow Transplantation, Aichi Medical University School of Medicine, Nagakute, Japan; 2 St. Vincent’s Pathology, St. Vincent’s Health Network Sydney, Australasian Bone Marrow Transplant Recipient Registry (ABMTRR), Sydney, Australia; 3 Department of Haematology, Singapore General Hospital, Singapore, Singapore; 4 Australasian Bone Marrow Transplant Recipient Registry (ABMTRR), Sydney, Australia; 5 Department of Hematology & BMT, Dhaka Medical College & Hospital, Dhaka, Bangladesh; 6 Department of Bone Marrow Transplantation, Ludaopei, Hematology & Oncology Center, Yanda International Hospital, Hebei, China; 7 Department of Medicine, Queen Mary Hospital, University of Hong Kong, Hong Kong, Hong Kong; 8 Hematology, Oncology and Stem Cell Research Center, Tehran University of Medical Sciences, Tehran, Iran; 9 Department of Pediatrics, Seoul National University College of Medicine, Seoul National University Cancer Research Institute, Seoul, South Korea; 10 Department of Haematology, Ampang Hospital, Selangor Darul Ehsan, Malaysia; 11 Department of Clinical Haematology, North Okkalapa General Hospital, Yangon, Myanmar; 12 Department of Clinical Haematology, National Institute of Blood Disease & Bone Marrow Transplantation, Karachi, Pakistan; 13 Blood and Marrow Transplant Center, St. Luke’s Medical Center, Quezon City, Philippines; 14 Faculty of Medicine, Sri Lanka and Central Hospital, University of Colombo, Colombo, Sri Lanka; 15 Department of Internal Medicine, National Taiwan University Hospital, Taipei, Taiwan; 16 Research Collaborations in Hematologic Malignancies and Hematopoietic Stem Cell Transplantation, Division of Hematology, Chulalongkorn University, Bangkok, Thailand; 17 Stem cell transplantation department, Blood Transfusion and Hematology Hospital, Ho Chi Minh, Vietnam; 18 Department of Haematology, Christian Medical College Hospital, Vellore, India; 19 Division of Hematology, Department of Medicine, Keio University School of Medicine, Tokyo, Japan; 20 Japanese Data Center for Hematopoietic Cell Transplantation (JDCHCT), Nagoya, Japan

**Keywords:** Epidemiology, Haematological diseases

## Abstract

Between 2005 and 2015, 138,165 hematopoietic stem cell transplantation (HSCT) were reported in 18 countries/regions in the Asia-Pacific region. In this report, we describe current trends in HSCT throughout the Asia-Pacific region and differences among nations in this region and various global registries. Since 2008, more than 10,000 HSCTs have been recorded each year by the Asia-Pacific Blood and Marrow Transplantation Group Data Center. Between 2005 and 2015, the greatest increase in the number of HSCTs was observed in Vietnam. Allogeneic HSCT was performed more frequently than autologous HSCT, and a majority of cases involved related donors. Regarding allogeneic HSCT, the use of cord blood has remained steady, especially in Japan, and the number of cases involving related HLA non-identical donors has increased rapidly, particularly in China. The incidence of hemoglobinopathy, a main indication for allogeneic HSCT in India, China, Iran, and Pakistan, increased nearly six-fold over the last decade. Among the 18 participating countries/regions, the transplant rate per population varied widely according to the absolute number of HSCTs and the national/regional population size. We believe that this report will not only benefit the AP region but will also provide information about HSCT to other regions worldwide.

## Introduction

Hematopoietic stem cell transplantation (HSCT) is the curative treatment modality of choice for many malignant and non-malignant hematologic disorders, and the annual global frequency of this procedure has increased steadily over the past decade [1]. In 2010, Yoshimi et al. published the first report of the number of HSCTs performed between 1986 and 2006 in nine countries/regions in the Asia-Pacific (AP) region [2]. Since then, the Asia-Pacific Blood and Marrow Transplantation Group (APBMT) Data Center has continuously collated the HSCT cases reported in each participating country. The APBMT Activity Survey, which comprises items regarding the HSCT type, donor type, stem cell source, and disease type, is used for collation. Subsequently, the survey data are used to analyze the latest trends in HSCT throughout the AP region, promote HSCT in both emerging economies and advanced countries, and collaborate on various international research studies.

In the last decade, the options for donor selection and the disease indications for HSCT have been expanded [1]. This report aims to summarize the APBMT data and compare the findings to those from Western countries with the intent to identify trends in HSCT in the AP region.

## Materials and methods

This detailed retrospective analysis was based on APBMT Activity Survey Data collected from 2005 to 2015. We set 2005 as the earliest time point because some newly participating countries/regions in the APBMT Activity Survey (Australia and New Zealand) had already reported activities prior to the publication of our first report [2]. We also used partial data accumulated since 1986 to summarize activity in this region. At the end of December 2017, 18 of 21 APBMT member countries/regions had submitted data from 2005 to 2015 to the APBMT Data Center.

Six countries/regions (Australia, New Zealand, India, Republic of Korea [referred to as Korea in this paper], Japan, and Taiwan) have their own national registries: the Australasian Bone Marrow Transplant Recipient Registry (ABMTRR), Indian Stem Cell Transplant Registry (ISCTR), Korean Society of Blood and Marrow Transplantation (KSBMT), Japan Society for Hematopoietic Cell Transplantation (JSHCT)/Japanese Data Center for Hematopoietic Cell Transplantation (JDCHCT), and Taiwan Society of Blood and Marrow Transplantation (TBMT), respectively. These countries/regions submitted national registry data every year. In China, Iran, Malaysia, and the Philippines, a single contact person from a major transplant center collated the number of entire HSCTs performed in their country/region each year and submitted those data (subsequently, national registry systems were created in China and Malaysia). Hospitals or centers in Bangladesh, Hong Kong, Myanmar, Pakistan, Singapore, Sri Lanka, Thailand, and Vietnam sent data individually to the APBMT Data Center, which aggregated these data by country. By the end of 2017, the APBMT Data Center had not received data from Cambodia, Indonesia, and Mongolia, although certain HSCT activities were known to have occurred in Indonesia and Mongolia.

The number of HSCTs was collated according to HSCT type, donor type, stem cell source, and disease type. If one patient underwent two transplants in 1 year, the APBMT counted this case as two HSCTs. HSCTs involving multiple stem cell sources during a single procedure were conventionally counted as one HSCT and categorized as a multiple stem cell source transplant; these cases included bone marrow (BM) plus peripheral blood stem cells (PB), BM plus cord blood cells (CB), PB plus CB, or BM plus PB plus CB. Although the APBMT Data Center had not previously used the word “haploidentical”, we considered the term “HLA non-identical family” to be roughly synonymous to “haploidentical.” Among the disease indications, this survey classified thalassemia, sickle cell disease, and other hemoglobinopathies as hemoglobinopathies.

To compare trends in HSCT over time, the rate of increase was calculated by dividing the frequency of HSCT during 2015 by the frequency in 2005. The transplant rates in each country/region were calculated as the number of each type of HSCT per 10 million residents in 2015. The total population for each country was extracted from a United States Census Bureau report [3], and the gross domestic product (GDP) and GDP per capita were extracted from World Bank data [4]. All analyses in this report were conducted at the APBMT Data Center. The present survey was approved by the institutional review board of the Aichi Medical University School of Medicine.

## Results

### Overview of trends in HSCT and center numbers

Eighteen of 21 APBMT member countries/regions reported their annual HSCT activities to the APBMT Data Center every year between 2005 and 2015 (Table 1). Centers in Bangladesh, Myanmar, and Sri Lanka began performing HSCTs in 2014 after their initial participation in the Worldwide Network for Blood and Marrow Transplantation/World Health Organization (WBMT/WHO) Workshop in Hanoi, 2011 [5]. The total number of centers in the AP region in 2015 was 624, and the numbers of centers varied by country/region, ranging from one center each in Bangladesh, Myanmar, and Sri Lanka to 373 centers in Japan.

**Table 1:** Annual number of HSCTs performed in each country/region of the Asia-Pacific region from 2005 to 2015, and the number of centers in 2007 and 2015

Countries/regions	2005	2006	2007	2008	2009	2010	2011	2012	2013	2014	2015	Total No. of HSCTs performed from 2005 to 2015	Ratio of 2015/2005^b^	No. of Centers	
													2015/2006^a^	2007^c^	2015
Australia	1201	1127	1175	1209	1327	1389	1450	1567	1509	1625	1723	15,302	1.4	41	41
Bangladesh										9	8	17	NA		1
China	459	399	1203	1604	1417	1732	1910	3140	3402	4207	4052	23,525	8.8	33	64
Hong Kong	142	142	169	133	149	160	176	131	115	139	139	1595	1	2	2
India		266	295	409	562	599	877	932	1112	1474	1636	8162	6.2^a^	15	41
Iran	279	325	364	389	366	491	451	624	431	435	438	4,593	1.6	2	9
Japan	3748	4062	4065	4204	4425	4807	4924	5364	5291	5458	5609	51,957	1.5	359	373
Korea	1139	1315	1382	1459	1672	1773	1900	1930	2012	2224	2286	19,092	2	37	44
Malaysia	147	124	135	181	213	262	271	303	312	334	401	2,683	2.7	10	9
Myanmar										1	2	3	NA		1
New Zealand	149	147	115	171	201	205	201	245	235	228	260	2157	1.7	6	6
Pakistan	58	76	80	94	106	100	109	108	115	89	143	1078	2.5	2	3
The Philippines	4	4	3	3	2	4	7	11	7	15	28	88	7		2
Singapore	136	121	130	124	133	155	164	149	157	188	182	1639	1.3	3	5
Sri Lanka										4	9	13	NA		1
Taiwan	203	381	381	337	388	467	492	458	477	459	521	4564	2.6	8	18
Thailand	100	127	133	131	163	131	72	133	213	118	57	1378	0.6	5	2
Vietnam	6	5	9	19	11	4	6	57	47	77	78	319	13	2	2
Total	7771	8621	9639	10,467	11,135	12,279	13,010	15,152	15,435	17,084	17,572	138,165	2.3	525	624

*HSCT* hematopoietic stem cell transplantation

^a^The official number of HSCTs performed in India since 2006

^b^Ratio of number of HSCTs performed in 2015 to that in 2005

^c^The official number of HSCT centers in APBMT since 2007

The annual number of HSCTs performed in the AP region has increased continuously each year. The annual number has exceeded 10,000 each year since 2008, and nearly 200,000 total HSCTs were performed from 1986 [2] to 2015. A comparison of changes in the rates of HSCTs from 2005 to 2015 revealed the greatest increase in Vietnam (13.0), followed by China (8.8), the Philippines (7.0), India (6.2), Malaysia (2.7), Taiwan (2.6), and Pakistan (2.5) (Table 1).

### Allogeneic vs. autologous transplantation

**Fig. 1:**
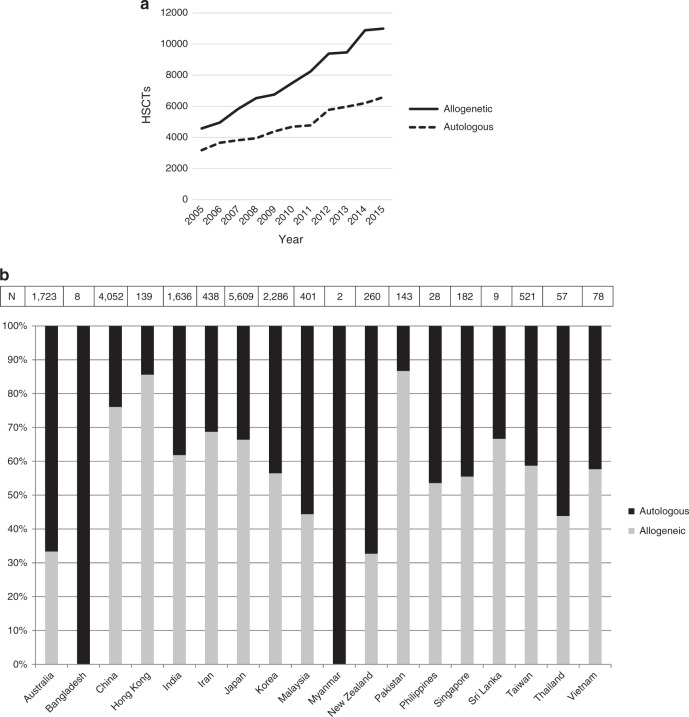
**a** Number of hematopoietic stem cell transplants (HSCTs) performed between 2006 and 2015 by transplant type. **b** Proportions of autologous and allogeneic HSCTs performed in 2015 by country/region

Allogeneic HSCT was performed more frequently than autologous HSCT, and the gap between these transplant types increased each year (Fig. 1a). The proportion of allogeneic HSCTs ranged widely among the 18 registered countries/regions, from 32.7% in New Zealand to 86.7% in Pakistan. Only four countries/regions recorded more cases of autologous HSCT vs allogeneic HSCT in 2015 (Australia, Malaysia, New Zealand, and Thailand). Bangladesh and Myanmar performed no allogeneic HSCTs in 2015, which was attributed to the initiation of HSCT after participation in the 1st WBMT/WHO Workshop in 2011 and observation of the recommendations made by the WBMT [6] (Fig. 1b).

### Donor types and stem cell sources

**Fig. 2:**
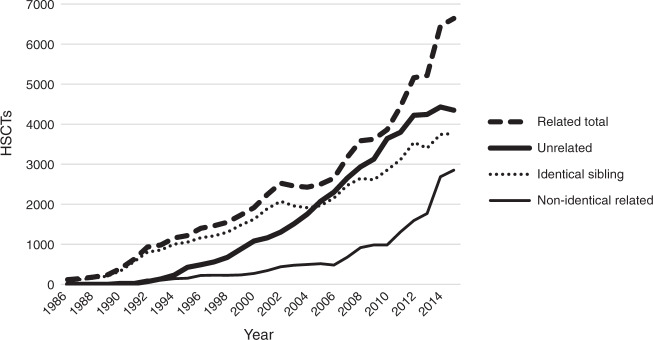
Trends in the number of hematopoietic stem cell transplants (HSCTs) by donor type

As shown in Fig. 2, which comprises data compiled during the first report [2], the number of HSCTs with related donors was consistently higher than the number of HSCTs with unrelated donors. Although the gap in the frequencies of these two donor types decreased from 2004 to 2011, it began to increase in 2014. In nine of 16 countries/regions (56%), more than 80% of HSCTs involved related donors. Australia, Japan, and New Zealand were the only countries that performed more HSCTs involving unrelated donors (Table 2). An analysis of the main donor types in allogeneic transplantation cases revealed a recent and rapid increase in the number of HSCTs from HLA non-identical related donors, compared to the mild increases in the numbers of HSCTs from identical siblings and unrelated donors.

**Table 2:** Number of HSCTs performed according to stem cell source in 2015

	Related				Unrelated						
	BM	PB	CB	Multiple	BM	PB	CB	Multiple	Related/Unrelated	BM Bank	CB Bank
Australia	40	181	1	0	57	255	41	0	0.6	+	+
Bangladesh	0	0	0	0	0	0	0	0	NA	−	−
China	26	1082	34	1369	1	439	125	7	4.4	+	+
Hong Kong	25	36	0	1	24	27	6	0	1.1	+	+
India	144	775	1	15	3	64	10	0	12.1	+	+
Iran	16	263	2	0	4	16	0	0	14.1	+	+
Japan	301	932	0	10	1175	55	1251	0	0.5	+	+
Korea	63	689	0	3	8	475	53	0	1.4	+	+
Malaysia	28	124	0	2	9	14	1	0	6.4	+	+
Myanmar	0	0	0	0	0	0	0	0	NA	−	−
New Zealand	4	35	0	0	5	33	8	0	0.8	+	+
Pakistan	72	37	0	15	0	0	0	0	NA	−	−
The Philippines	0	15	0	0	0	0	0	0	NA	−	−
Singapore	5	58	0	0	2	26	10	0	1.7	+	+
Sri Lanka	6	0	0	0	0	0	0	0	NA	−	−
Taiwan	5	142	0	23	3	130	3	0	1.3	+	+
Thailand	1	18	0	3	0	3	0	0	7.3	−	+
Vietnam	0	38	1	0	0	0	6	0	6.5	+	+
Total	736	4425	39	1441	1291	1537	1514	7	1.5		

*BM* bone marrow, *PB* peripheral blood, *CB* cord blood, *HSCT* hematopoietic stem cell transplantation

An analysis of the stem cell sources for allogeneic HSCT demonstrated that the number of peripheral blood stem cell transplants (PBSCT) first overtook that of BM transplants (BMT) in 2007. Subsequently, the frequency of PBSCT has increased continuously in the AP region. Furthermore, the number of HSCTs with multiple stem cell sources has increased over the past years, and 95.0% of these cases were performed in China in 2015 (Table 2). The most popular multiple stem cell source combination was BM plus PB from a human leukocyte antigen (HLA) non-identical family member (60.4%). Furthermore, that among cases with unrelated donors, the number of cord blood transplants (CBT) was equivalent to the total number of cases involving BM and PB in Japan (Table 2).

Notably, 82.6% of all CBTs were performed in Japan. The high frequency of PBSCT in cases of non-malignant diseases was also a characteristic trend observed in the Asia-Pacific region (Table 3).

**Table 3:** Number of HSCTs by stem cell sources in allogeneic HSCT (malignant diseases vs non-malignant diseases)

	BM	PB	CB	Multiple
Malignant diseases	1560 (17.0%)	5021 (54.6%)	1403 (15.3%)	1208 (13.1)
Non-malignant diseases	456 (25.3%)	939 (52.1%)	170 (9.4%)	239 (13.2%)

*BM* bone marrow, *PB* peripheral blood, *CB* cord blood, *HSCT* hematopoietic stem cell transplantation

### Indications

**Fig. 3:**
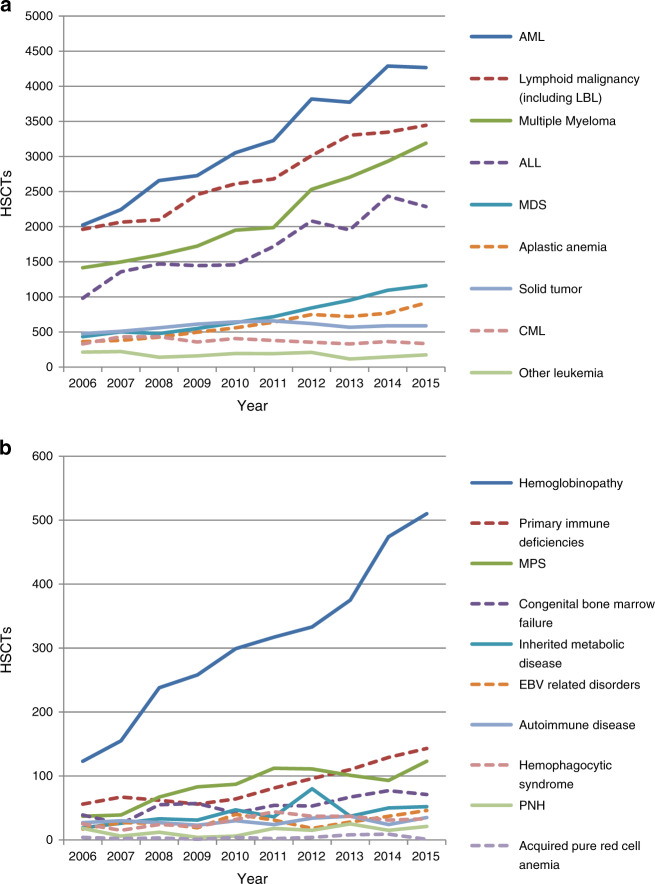
The number of hematopoietic stem cell transplants (HSCTs) by disease. **a** Diseases associated with more than 200 HSCTs or **b** fewer than 200 HSCTs in 2006

The HSCT numbers increased steadily from 2005 to 2015 for all diseases except chronic myelogenous leukemia (CML) (Fig. 3a, b). In 2015, acute myelogenous leukemia (AML) accounted for 24.3% of all HSCTs, followed by lymphoid malignancies (19.6%), plasma cell disorders (18.1%), and acute lymphoid leukemia (ALL) (13.0%). Although hemoglobinopathy accounted for only 2.9% of all HSCTs, the rate of increase in this disease was prominent, compared to those of other major diseases (Fig. 3b). During the past decade, the number of HSCTs performed for hemoglobinopathy increased by more than six-fold, and these procedures were performed in India, China, Iran, Pakistan, Korea, and Malaysia. The numbers of HSCTs performed for CML decreased in 2015 relative to early 2000s in all countries, except India and Malaysia.

### Transplant rates

**Fig. 4:**
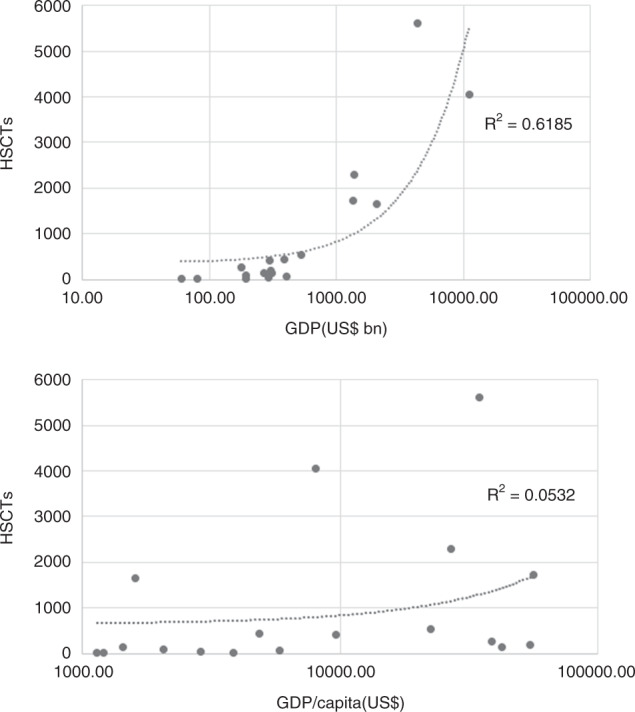
Correlation between absolute numbers of transplants and the gross domestic product (GDP, top) and (bottom)

As shown in Table 4, the rates of each type of transplant per population varied widely among the AP countries/regions. Figure 4 depicts the relationships between the absolute number of transplants and GDP/GDP per capita. As shown, the transplant numbers correlated with GDP rather than GDP per capita in the AP region.

**Table 4:** Transplant rate of each type of HSCT and team density per 10 million population in each country/region in 2015

Country/region	Population (millions)	Total HSCT	Allogeneic HSCT	Autologous HSCT	Unrelated HSCT	Team density
Australia	24.1	714.9	238.6	476.3	146.5	17.0
Bangladesh	162.9	0.5	0	0.5	0	0.1
China	1403.5	28.9	22	6.9	4.1	0.5
Hong Kong	7.3	190.4	163	27.4	78.1	2.7
India	1324.2	12.4	7.6	4.7	0.6	0.3
Iran	80.3	109.1	92	17.1	2.5	1.1
Japan	127.7	439.2	291.6	147.6	194.3	29.2
Korea	50.8	450	254.1	195.9	95.1	8.7
Malaysia	31.2	128.5	57.1	71.5	7.7	2.9
Myanmar	52.9	0.4	0	0.4	0	0.2
New Zealand	4.6	565.2	184.8	380.4	100	13.0
Pakistan	193.2	7.4	6.4	1	0	0.2
Philippines	103.3	2.7	1.5	1.3	0	0.2
Singapore	5.6	325	180.4	144.6	67.9	8.9
Sri Lanka	20.8	4.3	2.9	1.4	0	0.5
Taiwan	23.5	221.7	130.2	91.5	57.9	7.7
Thailand	68.9	8.3	3.6	4.6	0.4	0.3
Vietnam	94.6	8.2	4.8	3.5	0.6	0.2

## Discussion

After Thomas et al. reported comprehensive studies that aimed to obtain successful HSCT outcomes [7, 8], some HSCTs were performed in AP region even in the 1970s [9, 10]. However, HSCT was not applied regularly until the 1980s [11–15]. In 2007, the APBMT Data Center began to survey HSCT activity in related to procedures performed since 1986. Since the initial APBMT report of HSCT activities published in 2010 [2], the number of countries/regions reporting their annual data has doubled (from 9 to 18), and the number of HSCT centers increased by 43.8% (from 432 to 621). In 2015, 17,572 HSCTs were performed, representing a 2.3-fold increase in the number of HSCTs performed (from 7771 to 17,572) since 2005. Among the 18 participating countries/regions, the increases in the numbers of reported HSCTs were particularly noticeable in Vietnam, China, the Philippines, and India. These increases were attributed to (1) the establishment and organization of academic activities and international collaborations through the annual APBMT Congresses and WBMT workshops [5] in Vietnam [16] and the Philippines [17] after the late 2000s, and (2) the very large national populations, recent remarkable economic growth, and advances in medical services in China and India [3, 18].

Our results demonstrate the diversity of HSCT activities throughout the AP region. Three countries have just implemented HSCT programs (Bangladesh, Myanmar, and Sri Lanka), while three others have not yet reported HSCT activities (Cambodia, Indonesia, and Mongolia). By contrast, Japan, Korea and Australia have consistently reported more than 1000 HSCTs performed annually since 2005. The number of HSCT centers is extremely high in Japan because HSCT is routinely performed by hematology departments, mostly due to high coverage of the national health insurance scheme. The fluctuations in the number of HSCTs performed in Thailand might result from insufficient communication among the domestic centers in Thailand as well as our data center. Despite the huge disparities in socioeconomic statuses among countries in the AP region [4], HSCT has increased steadily in all member countries of the APBMT in the past decade. Nevertheless, these huge disparities mean that the data in this report may not necessarily reflect all regional trends, but rather the trends of the few countries participating in the survey. Gratwohl et al. reported an association of the economic gap between low-income and high-income countries/regions with global transplant rates and suggested that increased regional support would foster the growth of HSCT programs [19].　To overcome the potential effects of this disparity, we have specified the names of the corresponding countries/regions where necessary.

An overview of the observed trends reveals a remarkable difference between the data from Western countries and those from the AP region. Namely, allogeneic HSCTs were performed more frequently than autologous HSCT in the AP region. Gratwohl et al. reported that on average allogeneic HSCTs accounted for 38 and 46% of all HSCTs performed in Europe and the Americas, respectively, during 2006–2012 [1, 20]. In the AP region, however, 12 of 16 participating countries/regions reported that allogeneic HSCT was performed more frequently than autologous HSCT (Table 2), with allogeneic HSCTs accounting for 63.5% of all procedures in 2015 (excluding data from Bangladesh and Myanmar, which had not yet initiated allogeneic transplantation in 2015). We note that the HSCT rates per population in India and China remained relatively low, despite the relatively large absolute numbers of HSCTs in these countries. Although we might attribute this discrepancy to the large populations of these countries, the absolute numbers of procedures correlated strongly with the GDP of each country.

Our study revealed three characteristic findings regarding donor types and stem cell sources. First, we elucidated the number of PBSCTs from unrelated donors and CBTs in Japan. Although the Japan Marrow Donor Program (JMDP) facilitated large numbers of unrelated HSCTs using BM cells and the number of PBSCTs from related donors was also large, the number of PBSCTs from unrelated donors was markedly small in comparison (Table 2). We attribute this finding to the specific JMDP facilitation of G-CSF-mobilized peripheral blood harvesting from volunteer donors after a 10-years project confirming the safety of family donors [21]. Accordingly, Japan launched an unrelated PBSCT program in 2010. The cumulative number of PBSCTs from unrelated donors remained small at the end of 2015 due to stepwise increases in the performance of this procedure [22]. By contrast, the number of CBTs performed in Japan was large, consistent with the cumulative annual numbers of HSCTs with related or unrelated donors, and increased continuously. Uchida et al. reported that nearly a third of all CBTs worldwide were performed in Japan [23, 24], contrary to a recent global decreasing trend in the performance of CBT and concomitant increase in HSCTs from haploidentical donors [25, 26]. This discrepancy may be attributable to the relatively small body size of an adult patient; in most cases, therefore, a single unit of CB stem cells can provide sufficient cell numbers for engraftment. Currently, the six Japan Cord Blood Banks aim to collect more than 10^9^/units of CB stem cells, which would cover the majority of adult recipients.

Second, China reported a large number of HSCTs from related donors that involved the co-infusion of stem cells from multiple sources (Table 2). Based on the long-term experiences with haplo-HSCT according to the Beijing Protocol, Lu, Huang, and colleagues proposed that graft-versus-host disease (GVHD) and rejection after haplo-HSCT could be prevented by the co-infusion of G-CSF-mobilized BM and PB and the administration of anti-thymocyte globulin [27, 28]. In this region, the relatively large numbers of HSCTs from HLA non-identical family donors are mostly attributable to Chinese cases and may reflect the difficulty of identifying an HLA-matched sibling donor due to the recently recalled single-child policy. Accordingly, China simultaneously developed a HSCT bank system for BM, PB, and CB and a haploidentical transplant technique that differs from the current Post-transplant cyclophosphamide (PT/CY) used in Western countries [29].

Third, the number of cases using PB as a stem cell source overtook those using BM, even for non-malignant diseases (Table 3). This trend is largely attributable to India and differs from the patterns observed in the US and Europe [30]. In India, PBSCT is preferentially selected to improve engraftment in patients with non-malignant diseases under non-radiation regimens (from a personal communication with A Srivastava). We await the outcomes of this trend in India.

The last decade saw increases in the number of HSCTs performed for each disease except for CML. Furthermore, each hematological malignancy was reported as a top indication for HSCT in all AP countries/regions, consistent with reports from other regions [26, 31]. Nevertheless, the reported incidences of plasma cell disorders and lymphoid malignancies, the major targets of autologous transplantation, were lower in the AP region than in Western countries [26]. Most APBMT member countries may place a greater priority curative allogeneic transplantation for leukemia and MDS than on prolonging the survival of patients with plasma cell disorders or lymphoid malignancies. Similar trends have been observed for non-malignant hematological diseases. In this context, hemoglobinopathy was among the most important indications for allogeneic HSCT, and the rate of increase in the number of transplants for this disease was remarkably higher than that of other diseases (Fig. 3b). In summary, the definitive indication of allogeneic HSCT for hemoglobinopathy and primary indication of this procedure for leukemia/MDS might partly explain the dominance of allogeneic HSCT in this region.

In conclusion, we observed a wide range of HSCT activities in the AP region. Notably, wide variations were observed in the transplant and donor types, stem cell sources, and disease indications among the member countries/regions. Notably, both the number of AP countries/regions with centers performing HSCT and the number of HSCTs have increased in the past decade. We believe that the data in this report will not only be useful to AP countries/regions but will also inform other regions worldwide [20].

## Appendix

All contributing centers that were registered with the APBMT Data Center are listed here (some centers may no longer perform HSCT, may have merged with other centers, or changed their names).

*Australia*

Alfred Hospital, Ashford Cancer Center, Box Hill Hospital, Brisbane Private Hospital, Canberra Hospital, Concord Hospital, Fremantle Hospital, Geelong Hospital, Gosford Hospital, Greenslopes Private Hospital, John Hunter Children’s Hospital, Liverpool Hospital, Mater Hospital Brisbane, Mater Private Hospital Brisbane, Nepean Hospital, Newcastle Mater Hospital, Peter MacCallum Cancer Center, Prince of Wales Hospital, Princess Alexandra Hospital, Princess Margaret Hospital for Children, Queen Elizabeth Hospital, Royal Adelaide Hospital, Lady Cilento Children’s Hospital, Royal Brisbane Hospital, Royal Children’s Hospital Melbourne, Royal Hobart Hospital, Royal Melbourne Hospital, Royal North Shore Hospital, Royal Perth Hospital, Royal Prince Alfred Hospital, Sir Charles Gairdner Hospital.

St George Hospital, St Vincent’s Hospital Sydney, St Vincent’s Hospital Melbourne, Sydney Children’s Hospital, The Children’s Hospital at Westmead, Townsville Hospital, Wesley Clinic, Westmead Hospital, Wollongong Hospital, Women & Children’s Hospital, Austin Hospital, Flinders Medical Center, Fiona Stanley Hospital, Gold Coast University Hospital.

*Bangladesh*

Dhaka Medical College & Hospital.

*China (mainland)*

Beijing Cancer Hospital, Beijing Chao-Yang Hospital, Ludaopei Hematology & Oncology Center, Beijing Hospital, Cancer Institute & Hospital, Chinese Academy of Medical Sciences, The First Affiliated Hospital of Zhejiang University, The First Affiliated Hospital of Zhengzhou University, The First Affiliated Hospital of China PLA General Hospital, The First Affiliated Hospital of China PLA General Hospital, Hainan No.1 Provincial People’s Hospital, Henan Institute of Hematology, Cancer Hospital of Henan, Huashan Hospital, Jiangsu Institute of Hematology,The First Affiliated Hospital of Soochow University, Jiangsu Province Hospital,The First Affiliated Hospital of Nanjing Medical University, Nanjing Drum Tower Hospital, Peking University First Hospital, Peking University People’s Hospital, Shanghai Changzheng Hospital, Shanghai Children’s Medical Center, Shanghai Daopei Hospital, Shanghai Ruijin Hospital, The Third Affiliated Hospital of Sun Yat-sen University, Tongren Hospital, Union Hospital Fujian Medical University, Union Hospital Tongji Medical College of Huazhong University of Science and Technology, West China Hospital of Sichuan University, Xinhua Hospital Affiliated to Shanghai Jiaotong University School of Medicine, Xuanwu Hospital Capital Medical University, Beijing Friendship Hospital, Zhongshan Hospital of Xiamen University, Zhujiang Hospital Southern Medical University, Chinese PLA General Hopital, Harbin Hematology and Cancer Institution, Nanfang Hospital Southern Medical University, PLA Navy General Hospital, PLA. The Military General Hospital of Beijing, Tangshan Iron and Steel Company Hospital, The First Affiliated Hospital of Guangxi Medical University, The Second Affiliated Hospital of Henan Medical University, Xinqiao Hospital of the Third Military Medical University, 309th Hospital of PLA, Changhai Hospital of Shanghai, Xinjiang Uygur Autonomous Region People’s Hospital, Zhejiang Provincial Hospital of Traditional Chinese Medicine, The First Affiliated Hospital of Medical College of Xi’an Jiao Tong University, Shandong Provincial Hospital, Hospital of Guizhou Medical University, Guangdong General Hospital, Xiangya Hospital Central-South University, The First Hospital of China Medical University, Anhui Provincial Hospital, Shangdong Provincial Qianfoshan Hospital, CAMS & PUMC, Hainan General Hospital, Kunming General Hospital of PLA, Ludaopei Hematology & Oncology Center, Ningbo First Hospital, Peking University Third Hospital, Qilu Hospital of Shandong University, The Affiliated Hospital of Qingdao University, The First Affiliated Hospital of Fujian Medical University, The Second Artillery General Hospital of Chinese PLA, The Second Hospital of Hebei Medical University, Tianjin First Central Hospital, Tianjin Medical University Cancer Institute and Hospital, West China Women’s and Children’s Hospital, Xijing Hospital, He Nan Provincial People’s Hospital, Fujian Provincial Hospital, Shengjing Hospital of China Medical University, Sun Yat-sen Memorial Hospital,Sun Yat-sen University, The First Affiliated Hospital of Dalian Medical University, Yanda International Hospital, Ludaopei Hematology & Oncology Center, Jinan Military General Hospital, The First Affiliated Hospital of He Nan Science & Technology University, The General Hospital of Shenyang Military, Tongji Hospital, Huazhong University of Science & Technology, Anhui Provincial Hospital, Nanfang Hospital Southern Medical University(Nan Fang Hospital (Adults), Changhai Hospital of PLA, Xiehe Hospital of Beijing, The First Affiliated Hospital of Xinjiang Medical Collage, The First Hospital of Shanghai, The First Affiliated Hospital of suzhou University, Hematology Department of 307 Hospital of Hematology Department of PLA, Zhejiang Provincial Hospital of Traditional Chinese Medicine, The First Affiliated Hospital of Wenzhou (Medical) University, Ruijin Hospital.

*Hong Kong*

Queen Mary Hospital, The University of Hong Kong, Prince of Wales Hospital, The Chinese University of Hong Kong.

*India*

Christian Medical College, Army Research & Referral Hospital, Tata Memorial Center, All India Institute of Medical Science, Sahyadri Specialty Hospital, Jaslok Hospital and Research Center, Narayana Multi specialty Hospital and Mazumdar Shaw Cancer Center, Gujarat Cancer & Research Institute, Rajiv Gandhi Cancer Institute & Research Center, Postgraduate Institute of Medical Education & Research, Manipal Hospital, Prince Aly Khan Hospital, Ruby Hall Clinic, Sir Ganga Ram Hospital, Christian Medical College, Apollo Cancer Specialty Hospital, St. John’s Medical College & Hospital, Netaji Subhaschandra Bose Cancer Research Institute, Sterling Hospitals, Deenanath Mangeshkar Hospital, BLK Super Specialty Hospital, G Kuppusamy Naidu Memorial Hospital, Bhailal Amin General Hospital, Institute Rotary Cancer Hospital, Sanjay Gandhi Post Graduate Institute of Medical Sciences, Apollo Hospital, Health Care Global Enterprises, Nil Ratan Sircar Medical College & Hospital, Columbia Asia Referral Hospital, Institute of Hematology & Transfusion Medicine Institute: Medical College, Cancer Institute (WIA), Sterling Hospital, Tata Medical Center, Kovai Medical College & Hospital, Lotus Hospital, Central India Institute of Hematology & Oncology, Global Hospital & Transplant Center, Global Hospital & Transplant Center, Max Super Specialty Hospital, Amrita Institute of Medical Sciences and Research Center, RK Birla Cancer Center, SMS Medical College & Hospital, Malabar Cancer Center, Fortis Memorial Research Institute, Artemis Hospital, Saroj Gupta Cancer Center & Research Institute, Indraprastha Apollo Hospitals, M S Ramaiah Medical College, HCG-MSR Cancer Center, Yashoda Hospital, Dharamshila Hospital and Research Center, Meenakshi Mission Hospital, Apollo Gleneagles Hospital, Sri Ramachandra Medical College and Research Institute, Max Super Specialty Hospital, Shalimar Bagh, Command Hospital Air Force, Armed Forces Medical College & Command Hospital, Jupiter Hospital, LTM Medical College, P D Hinduja Hospital and MRC, Kokilaben Dhirubhai Ambani Hospital, Sahara Hospital, Manipal Hospital.

*Iran*

Shariati Hospital, Namizi Hospital, Transplant Research Center, Shiraz University of Medical Sciences, Imam Khomeini Hospital, Afzalipour Hospital, Imam Khomeini Hospital, Amir Kola Hospital, Petroleum Industry Hospital, Taleghani Hospital, Mahak Hospital, Montaserieh Hospital, Sherkat Naft Hospital, Shafa hospital-Ahvaz.

*Japan*

Hokkaido University Hospital, Sapporo Hokuyu Hospital, Sapporo Medical University Hospital, Asahikawa Medical College Hospital, Asahikawa Red Cross Hospital, Teine Keijinkai Hospital, Sapporo City General Hospital, National Hospital Organization Hokkaido Cancer Center, Hakodate Municipal Hospital, Asahikawa City Hospital, Hokkaido Medical Center for Child Health and Rehabilitation, Asahikawa-Kosei general Hospital, Steel Memorial Muroran Hospital, Oji General Hospital, Hirosaki University Hospital, Aomori Prefectural Central Hospital, Iwate Medical University School of Medicine, Tohoku University Hospital, National Hospital Organization Sendai Medical Center, Miyagi Cancer Center, Miyagi Children’s Hospital, Ishinomaki Red Cross Hospital, Osaki Citizen Hospital, Akita University School of Medicine, Nakadoori General Hospital, Yamagata University School of Medicine, Yamagata Prefectural Central Hospital, Fukushima Medical University School of Medicine, Iwaki Kyoritsu General Hospital, Ohta General Hospital, Ohta Nishinouchi Hospital, Kita-Fukushima Medical Center, University of Tsukuba, Tsukuba University Hospital, Ibaraki Children’s Hospital, Tsukuba Memorial Hospital, Tsuchiura Kyodo General Hospital, Hitachi General Hospital, National Hospital Organization Mito Medical Center, KKR Suifu Hospital, Jichi Medical University Hospital, Dokkyo Medical University School of Medicine, Tochigi Cancer Center, Saiseikai Maebashi Hospital, Gunnma University Graduate School of Medicine, Gunma Children’s Medical Center, National Hospital Organization Nishigunma National Hospital, Gunma Prefectural Cancer Center, Saitama Prefectural Cancer Center, Saitama Medical University International Medical Center, National Defense Medical College, Saitama Children’s Medical Center, Saitama Medical Center-Saitama Medical University, Saitama Medical Center Jichi Medical University, Comprehensive Cancer Center-International Medical Center-Saitama Medical University, Chiba University Hospital, Chiba Children’s Hospital, Matsudo City Hospital, Kameda General Hospital, Jikei University-Kashiwa Hospital, Chiba Aoba Municipal Hospital, Japanese Red Cross Narita Hospital, National Cancer Center Hospital East, Teikyo University Chiba Medical Center, Juntendo University Urayasu Hospital, Nippon Medical School Chiba Hokusoh Hospital, National Cancer Center Hospital, The Institute of Medical Science-The University of Tokyo, Tokyo Metropolitan Cancer and Infectious Disease Center-Komagome Hospital, Nihon University of School of Medicine Itabashi Hospital, The Jikei University school of Medicine, Keio University School of Medicine, Tokyo Medical University Hospital, Tokyo Women’s Medical University, Showa University School of Medicine, Kyorin University School of Medicine, NTT Kanto Medical Center, University of Tokyo Hospital, Juntendo University School of Medicine, Nippon Medical School, Teikyo University School of Medicine, Tokyo Metropolitan Kiyose Children’s Hospital, Toho University Omori Medical Center, St. Luke’s International Hospital, National Center for Child, Health and Development, Toranomon Hospital, International Medical Center of Japan, Faculty of Medicine Hospital Tokyo Medical and Dental University, National Hospital Organization, Tokyo Medical Center, Tokyo Metropolitan Fuchu Hospital, Japanese Red Cross Medical Center, Saiseikai Central Hospital, Tokyo Metropolitan Geriatric Hospital, Yokohama City University Hospital, Kanagawa Cancer Center, St. Mariannna University School of Medicine, Tokai University School of Medicine,Kanagawa Children’s Medical Center,Yokohama City University Medical Center, Showa University Fujigaoka Hospital, St. Marianna University School of Medicine Yokohama City Seibu Hospital, Yokohama Municipal Citizen’s Hospital, Yokohama City Minato Red Cross Hospital, Federation of National Public Service Personnel Mutual Aid Associations, Toranomon Hospital, Kajigaya, Yokohama Minami Kyosai Hospital, Niigata University Medical and Dental Hospital, Niigata Cancer Center, Nagaoka Red Cross Hospital, Toyama Prefectural Central Hospital, Kurobe City Hospital, Toyama University Hospital, Kouseiren Takaoka Hospital, Toyama Red Cross Hospital, Kanazawa University Hospital, Kanazawa Medical University Hospital, Ishikawa Prefectural Central Hospital, University of Fukui Hospital, University of Yamanashi-Faculty of Medicine, Yamanashi Prefectural Central Hospital, Shinshu University School of Medicine, Nagano Children’s Hospital, Nagano Red Cross Hospital, Gifu University Graduate School of Medicine, Gifu Municipal Hospital, Gifu Red Cross Hospital, Hamamatsu University School of Medicine, Hamamatsu Medical Center, Shizuoka General Hospital, Seirei Hamamatsu General Hospital, Shizuoka Children’s Hospital, Shizuoka Red Cross Hospital, Shizuoka Saiseikai General Hospital, Sizuoka Cancer Center, Juntendo University Shizuoka Hospital, Shizuoka City Shimizu Hospital, Japanese Red Cross Nagoya First Hospital, Nagoya Daini Red Cross Hospital, Meitetsu Hospital, Nagoya University Graduate school of medicine, Nagoya Ekisaikai Hospital, National Hospital Organization-Nagoya Medical Center, Nagoya City University Hospital, Anjo Kosei Hospital, Konan Kosei Hospital, Fujita Health University School of Medicine, Aichi Cancer Center Hospital, Toyohashi Municipal Hospital, Aichi Medical University School of Medicine, Social Insurance Chukyo　Hospital, Nagoya Memorial Hospital, Toyota Memorial Hospital, Toyota Kosei Hospital, Nishio Municipal Hospital, Mie University Hospital, Yamada Red Cross Hospital, Suzuka Kaisei Hospital, Suzuka General Hospital, Shiga University of Medical Science, Shiga Medical Center for Children, Otsu Red Cross Hospital, Omihachiman Community Medical Center, Kyoto University Hospital, Kyoto First Red Cross Hospital, Kyoto Prefectural University of Medicine, Social Insurance Kyoto Hospital, Kyoto City Hospital, Aiseikai Yamashina Hospital, Kyoto Katsura Hospital, Kyoto second Red Cross Hospital, Osaka Medical Center For Cancer And Cardiovascular Diseases, Kinki University School of Medicine, Osaka University Hospital, Osaka City University Graduate School of Medicine, National Hospital Organization Osaka National Hospital, Children’s Medical Center-Osaka City General Hospital, Osaka Red Cross Hospital, Osaka Medical Center and Research Institute for Material and Child Health, Matsushita Memorial Hospital, Kishiwada City Hospital, Rinku General Medical Center Izumisano Municipal Hospital, Osaka Medical College Hospital, Fuchu Hospital, Kansai Medical University Hirakata Hospital, Sakai Hospital, Kinki University School of Medicine, Sumitomo Hospital, The Tazuke Kofukai Medical Research Institute Kitano Hospital, Nissay Hospital, Takatsuki Red Cross Hospital, Yodogawa Christian Hospital, KKR Otemae Hospital, PL General Hospital, Hyogo College of Medicine, Hyogo Children’s Hospital, Hyogo Cancer Center, Kobe City Medical Center General Hospital, Kobe University Hospital, Ashiya Municipal Hospital, Akashi Municipal Hospital, Kobe Central Hospital of Insurance, Hyogo Prefectural Nishinomiya Hospital, Shinko Hospital, Meiwa Hospital, Nara Medical University, Tenri Hospital, Takanohara Central Hospital, Kinki Daigaku Igakubu Nara Hospital, Wakayama Medical University, Japanese Red Cross Society Wakayama Medical Center, Insurance Social Kinan Hospital, Tottori Prefectural Central Hospital, Tottori University Faculty of Medicine, National Hospital Organization, Yonago Medical Center, Shimane Prefectural Central Hospital, Shimane University, Faculty of Medicine, Matuse Red Cross Hospital, National Hospital Organization Okayama Medical Center, Kurashiki Central Hospital, Okayama University Hospital, Kawasaki Medical School, Okayama Rosai Hospital, National Hospital Organization, Minami-Okayama Medical Center, Okayama Citizens’ Hospital, Hiroshima Red Cross Hospital &Atomic-bomb Survivors Hospital, Hiroshima University Hospital, National Hospital Organization Kure Medical Center, Hirosima-Nishi Medical Center, Cyugoku Central Hospital, Yamaguchi University School of Medicine, Shimonoseki Kosei General Hospital, Tokushima University Hospital, Tokushima Red Cross Hospital, Kagawa University Faculty of Medicine, Kagawa National Children’s Hospital, Takamatsu Red Cross Hospital, Kagawa Prefectural Central Hospital, Ehime Prefectural Central Hospital, Matsuyama Red Cross Hospital, National Hospital Organization, Shikoku Cancer Center, Ehime University Graduate School of Medicine, Kochi Medical School-Kochi University, Kochi Health Sciences Center, Kyushu University Hospital, Harasanshin Hospital, Hamanomachi Hospital, OUR LADY OF SNOW medical juridical corporation St. MARY’S HOSPITAL, Kokura Memorial Hospital, Kurume University School of Medicine, Fukuoka University School of Medicine, National Kyushu Cancer Center, University of Occupational and Environmental Health-Japan, National Hospital Organization Kyusyu Medical Center, Kitakyusyu Municipal Medical Center, Kyushu Kosei-nenkin Hospital, Iizuka Hospital, SAGA-KEN MEDICAL CENTER KOSEIKAN, Saga University Hospital, Nagasaki University Graduate School of Biomedical Sciences, Japanese Red Cross Nagasaki Genbaku Hospital, Sasebo City General Hospital, National Hospital Organization-Nagasaki Medical Center, National Hospital Organization-Kumamoto Medical Center, Kumamoto University School of Medicine, Oita University Faculty of Medicine, Oita Prefectural Hospital, Oita Kouseiren Tsurumi Hospital, Kyusyu University Hospital at Beppu, Miyazaki Prefectural Miyazaki Hospital, University of Miyazaki Hospital, Imamura Bun-in Hospital, Kagoshima University Medical and Dental Hospital, Kagoshima City Hospital, National Hospital Organization Kagoshima Medical Center, University of the Ryukyus Faculty of Medicine, Okinawa Prefectural Nanbu Medical Center and Children’s Medical Center, Heart Life Hospital, Okinawa Red Cross Hospital.

*Korea*

The Catholic University Seoul St. Mary’s Hospital, The Catholic University Daejeon St. Mary’s Hospital, The Catholic University Our Lady Mercy Hospital, The Catholic University St. Vincent’s Hospital, Gachon University Gil Hospital, Kyungpook National University Hospital, Gyeongsan National University Hospital, Kyung Hee University Hospital, Korea University Guro Hospital, Korea University Anam Hospital, Kosin University Gospel Hospital, National Cancer Center, Daegu Catholic University Hospital, Daegu Fatima Hospital, Dong-A University Hospital, Pusan National University Hospital, Seoul National University Hospital, Sungkyunkwan University Hospital, Soonchunhyang University Bucheon Hospital, Soonchunhyang University Seoul Hospital, Ajou University Hospital, Yonsei University Hospital, Yeungnam University Hospital, Ulsan University Asan Medical Center, Ulsan University Hospital, Wonkwang University Hospital, Korea Cancer Center Hospital, Ewha Womans Univesity Mokdong Hospital, Inje University Paik Hospital, Inha University Hospital, Chonnam National University Hwasun Hospital, Chonbuk National University Hospital, Chungnam National University Hospital, Pochon Univesity Bundang CHA Hospital, Hallym University Hospital, Hanyang University Hospital, Chosun University Hospital, Chung-Ang University Hospital, Inje University Haeundae Paik Hospital, Jeju Halla General Hospital, Keimyung University Dongsan Medical Center, Konkuk University Medical Center, Pusan National University Yangsan Hospital, Yonsei University Wonju Christian Hospital, Keimyung University Dongsan Medical Center.

*Malaysia*

Hospital Ampang, Kuala Lumpur, Hospital Kuala Lumpur, Gleneagles Medical Center, Penang, Lam Wah Ee Hospital, Sime Darby Medical Center, Hospital University Kebangsaan Malaysia, University Malaya Medical Center, Ampang Puteri Specialist Hospital, Hospital University Sains Malaysia, Hospital Pulau Pinang, Hospital Melaka.

*Myanmar*

North Okkalapa General Hospital.

*New Zealand*

Auckland Hospital, Christchurch Hospital, Palmerston North Hospital, Starship Hospital, Waikato Hospital, Wellington Hospital.

*Pakistan*

National Institute of Blood Diseases and Blood and Marrow Transplantation, The Aga Khan University Hospital, Armed Forces Bone Marrow Transplant Center.

*The Philippines*

St. Luke’s Medical Center-Quezon City, St. Luke’s Medical Center-Global City, The Medical City, Makati Medical Center.

*Singapore*

National University Hospital, Singapore General Hospital, KK Hospital Women’s and Children’s Hospital, National Cancer Center, Singapore.

*Sri Lanka*

The Central Hospital, National cancer Institute.

*Taiwan*

Taipei Veterans General Hospital, National Taiwan University Hospital, National Taiwan University Hospital, Kaoshiung Medical University Hospital, China Medical University Hospital, Chang-Gung Memorial Hospital—Linko, Changhua Christian Hospital, Tri Service General Hospital, Koo Foundation Sun Yat-Sen Cancer Center, Hualien Tzu Chi Hospital-Buddhist Tzu Chi Medical Foundation, Chang-Gung Memorial Hospital—Chiayi, Chia-Yi Christian Hospital, National Cheng Kung University Hospital, Taichung Veterans General Hospital, Chi-Mei General Hospital, Kaoshiung Veterans General Hospital, Chang-Gung Memorial Hospital—Kaohsiung, Far Eastern Memorial Hospital, Taipei Medical University-Shuang Ho Hospital.

*Thailand*

Faculty of Medicine Ramathibodi Hospital, King Chulalongkorn Memorial Hospital, Phramongkutklao Hospital, Prince of Songkla University Hospital, Faculty of medicine Siriraj Hospital, Naresuan University, SrinagarindHospital-Khon Kaen University.

*Vietnam*

Blood Transfusion and Hematology Hospital, Hue Regional Hematology & Blood Transfusion Center, National Institute of Blood Transfusion and Hematology, National Hospital of Pediatrics, Cho Ray Hospital, Nghe An oncology hospital, Central Military Hospital 108, Bach Mai Hospital.
